# Effectively Communicating About HIV and Other Health Disparities: Findings From a Literature Review and Future Directions

**DOI:** 10.3389/fcomm.2020.539174

**Published:** 2020-06

**Authors:** Susana Peinado, Katherine Treiman, Jennifer D. Uhrig, Jocelyn Coleman Taylor, Jo Ellen Stryker

**Affiliations:** 1 Center for Communication Science, RTI International, Durham, NC, United States,; 2 Prevention Communication Branch, Division of HIV/AIDS Prevention, Centers for Disease Control and Prevention, Atlanta, GA, United States

**Keywords:** HIV, disparities (health racial), health communication, message framing, stigma, stereotype, targeting, unintended adverse effects

## Abstract

Despite significant progress in the prevention and treatment of HIV, disparities in rates of infection remain among key groups in the United States, including blacks and African Americans; Hispanics/Latinos; and men who have sex with men (MSM). The U.S. Department of Health and Human Services’ initiative, *Ending the HIV Epidemic: A Plan for America*, calls for addressing HIV-related disparities and reducing stigma and discrimination associated with HIV. The goal of this literature review was to identify approaches for effectively communicating about health disparities across the HIV care continuum. We reviewed the literature to investigate strategies used to communicate health disparities and to identify potential unintended adverse effects resulting from this messaging. Messages about health disparities often target subgroups at higher risk and can be framed in a variety of ways (e.g., social comparison, progress, impact, etiological). Studies have examined the effects of message framing on the risk perceptions, emotional reactions, and behaviors of individuals exposed to the messaging. The evidence points to several potential unintended adverse effects of using social comparison framing and individual responsibility framing to communicate about health disparities, and visual images and exemplars to target messages to higher-risk subgroups. There is not yet a clear evidence-based approach for communicating about health disparities and avoiding potential unintended effects. However, we offer recommendations for communicating about HIV-related disparities based on our findings. Because we found limited literature that addressed our research questions in the context of HIV, we propose a research agenda to build an evidence base for developing effective messages about HIV-related disparities.

## INTRODUCTION

*Healthy People 2020* defines a health disparity as “a particular type of health difference between individuals or groups that is unfair because it is caused by social or economic disadvantage” (U.S. Department of Health and Human Services, 2008). Despite significant progress in the prevention and treatment of HIV in the United States, disparities remain in rates of infection among racial/ethnic minority groups, with black and African American (hereafter referred to as black) and Hispanic/Latino populations being the most affected subgroups (McCree et al., 2017; U.S. Department of Health and Human Services, 2020). Gay, bisexual, and other men who have sex with men (MSM) are also disproportionately affected by HIV, and most MSM diagnosed with HIV are MSM of color. The causes of these disparities are complex and interrelated and can be attributed to multiple individual, social, contextual, and environmental factors (McCree et al., 2016).

### Ending the HIV Epidemic:

*A Plan for America* is the U.S. Department of Health and Human Services’ cross-agency initiative that aims to reduce new HIV infections in the U.S. by 90% in 10 years by focusing on communities most impacted by HIV (U.S. Department of Health and Human Services, 2020). This initiative highlights the persistence of HIV disparities among racial and ethnic minority groups as well as MSM and the role of stigma in preventing those at risk for HIV or living with HIV from receiving needed health care and services. HIV stigma may be exacerbated in marginalized groups who experience multiple and converging forms of stigma—referred to as intersectional stigma—including stigma related to race, ethnicity, sexual identity, gender identity or expression, illicit drug use, sex work, and incarceration (Earnshaw et al., 2013; Rice et al., 2018). Stigmatizing attitudes toward people with HIV or at risk for HIV may lead to delayed HIV testing (Golub and Gamarel, 2013), reduced adherence to antiretroviral therapy (Sweeney and Vanable, 2016), and poorer retention in care (Yehia et al., 2015). Social stigma can also have serious negative consequences for both psychological and physical well-being by decreasing self-esteem and increasing stress responses in stigmatized groups (Major and O’Brien, 2005).

Health communication can play a key role in raising awareness among priority audiences about their risk for getting or transmitting HIV and influencing attitudes, beliefs, and behaviors. However, communicating about health disparities can also result in unintended, adverse consequences. For example, dissemination of information comparing HIV diagnoses by subgroup may result in “blame and shame” and foster helplessness, distress, anger, and mistrust among affected communities (Friedman et al.’s, 2014; Lee et al., 2017; Drumhiller et al., 2018).

Smith (2007) developed a model of the social and psychological effects of messages that communicate stigma. According to Smith, stigma messages have four characteristics: (1) they distinguish or categorize a group of people, (2) they establish this group of people as a separate social entity, (3) they link the group to a physical or social threat, and (4) they imply that group members are responsible for the threat. Thus, stigma messages encourage stereotyping and the perception of the group as a coherent entity and make social identity salient (Major and O’Brien, 2005; Smith, 2007). These messages contribute to the perception of stigmatized individuals as a social threat and as responsible for their condition (Smith et al., 2019). Negative behavioral outcomes of exposure to stigma messages include increased support for interventions that isolate and regulate stigmatized groups, interpersonal disassociation from stigmatized individuals, and the social transmission of stigma messages (Smith et al., 2019). Because HIV and groups at greater risk for HIV are often associated with multiple social stigmas, communication about HIV and disparities in HIV are particularly susceptible to containing characteristics of stigma messages. Consequently, a better understanding of message strategies and features that raise awareness and motivate behavior change while avoiding potential adverse effects is needed.

We designed this literature review to be exploratory in nature. The overarching goal of the review was to identify promising approaches for effectively communicating about disparities across the HIV care continuum. As such, we reviewed the literature to address the following research questions (RQs):

1. What strategies are used to communicate health disparities information?
2. What are the potential unintended adverse effects of messages communicating health disparities, and how do specific message strategies contribute to these effects?

## METHODS

We searched four databases—PubMed, Web of Science, PsycINFO, and Communication Source—using the search terms shown in Table 1 for peer-reviewed literature published between 2011 and 2018. This time frame was selected to focus the review on the most recent literature that addressed our RQs. We also obtained additional articles *via* the snowball method, which involved reviewing the reference lists of particularly relevant articles and acquiring articles recommended by colleagues with subject matter expertise. Because the number of relevant articles identified by the database search was relatively small, we did not place any limitations on the time frame for articles obtained with the snowball approach.

We focused the search on studies conducted in the United States; however, we also included two articles from other countries because they examined RQs closely aligned with those of interest in this review. One study, identified by the snowball method, was conducted in South Korea (Lee and An, 2016) and described a randomized experiment testing the effects of messages about the controllability of a condition (i.e., individual responsibility for the onset of a condition) and group categorization on perceived stigma. The other study, conducted in the United Kingdom, reported results of interviews conducted to learn about the unintended consequences of an intervention targeting a high-risk group (Sorhaindo et al., 2016).

Although we prioritized literature focused on HIV, this body of literature was small. Consequently, we did not limit our search to HIV. We included relevant literature addressing our RQs across health topics, including sexually transmitted diseases (STDs), cancer, mental health, and obesity. Though some higher-risk populations may experience more layers of stigma than others, which could affect responses to messaging, we expected potential responses to messages about health disparities to be a communication phenomenon that would be similar across conditions, rather than entirely condition specific. Thus, we wanted to draw on the body of literature examining this phenomenon.

We were systematic in our approach to identifying relevant literature. However, the goal of this literature review was to be inclusive of relevant studies that addressed our RQs—including both quantitative and qualitative research—to gain an understanding the state of the science. Because the body of literature addressing our RQs was limited, we included a broader range of studies than would be included in a systematic review, which requires that studies meet certain specifications for design and quality. Broadening the body of literature we reviewed also allowed us to better synthesize literature at the intersection of our topics of interest and identify gaps in the existing literature.

We scanned titles and abstracts to identify potentially relevant articles and other documents, which resulted in 89 articles, book chapters, and reports for further review. On the basis of this review, we excluded sources that were not focused on our RQs, such as those that compared gain and loss frames rather than different strategies for framing disparities information. In total, we identified 43 articles and other documents from which we abstracted information that addressed the RQs.

## RESULTS

We found limited literature specifically addressing the RQs in the context of communicating HIV-related disparities (11 articles focused specifically on HIV and 3 on other STDs). Research assessing strategies and approaches used to communicate about health disparities often focused on cancer (e.g., Nicholson et al., 2008; Landrine and Corral, 2015), whereas much of the research about stigmatization and stereotyping focused on mental health (e.g., Corrigan et al., 2012, 2015), and obesity (e.g., Skurka, 2019). Only a few studies provided insights into how disparities in STDs and HIV can be presented to promote behavior change and avoid unintended adverse effects (Friedman et al.’s, 2014; Uhrig et al., 2017; Drumhiller et al., 2018).

We begin by describing strategies used to communicate health disparities. We then discuss potential unintended adverse effects that can result from the use of these strategies.

### Strategies for Communicating Health Disparities Information

Targeting and framing are communication strategies often used in messaging about HIV and other health disparities. In this section, we review literature that addresses our first RQ.

#### Targeting

Targeting, also referred to as audience segmentation, is a strategy used to increase the effectiveness of health messages and information (Slater, 1996). Targeting involves the decision to direct public health messages to a particular segment or segments of the population (i.e., priority audience), typically groups considered to be at “high risk” (Kreuter and Wray, 2003; Guttman and Salmon, 2004). The rationale for developing targeted messages is that they will better address the needs, concerns, beliefs, and values of a particular subgroup; increase the likelihood that the messages will be perceived as relevant; and promote positive behavior change (Slater, 1996; Institute of Medicine, 2002; Kreuter and Wray, 2003).

#### Message Framing

Message framing involves “select[ing] some aspects of a perceived reality and [making] them more salient in a communicating text” (Entman, 1993). Entman also describes frames as “defining problems,” “diagnosing causes,” “making moral judgments,” and “suggesting remedies.” The way information is framed is important because it has implications for how people view and understand the topic addressed in the communication (Entman, 1993). Frames can be used intentionally or unintentionally to communicate about health risks in public health messages and in the news media.

Framing is a strategy commonly used in messages about health disparities. We found that messages frame information about health disparities in a number of ways. *Social comparison framing* typically highlights disparities in disease incidence, risk, or outcomes between racial or other groups; for example, “Blacks are more than twice as likely as whites to be diagnosed with HIV” (Dunham et al., 2016). *Progress framing* highlights progress made in reducing health disparities, such as “Blacks Making Great Strides Against Colon Cancer” (Landrine and Corral, 2015). *Impact framing* presents the risks for one subgroup only (e.g., black only or white only). *Non-comparative framing* presents risks for the population overall (e.g., Americans; often used as a control in studies). *Etiological framing* or *causal framing* is when messages are framed to emphasize one or a combination of causal factors.

A small body of experimental studies examined the effects of message frames for communicating about health disparities on the risk perceptions, emotional reactions, behavioral intentions, and behaviors of priority audiences (i.e., those at higher risk) and those outside the priority audience. Table 2 provides an overview of these studies (*n* = 13) that address our first RQ. We discuss these studies in more detail below.

##### Social comparison framing

Several studies have examined the effects of presenting risk information in messages using a social comparison frame vs. messages using a non-comparative frame on participants’ risk perceptions, emotional reactions, and other outcomes (Uhrig et al., 2013; Bigman, 2014; Dunham et al., 2016; Jones et al., 2016; Skurka, 2019).

In a series of experiments, Bigman (2014) found that social comparison framing (comparing blacks and whites) of STD and cancer risk in mock news articles did not significantly raise risk perceptions among the group at higher risk (i.e., blacks in the case of STDs) relative to non-comparative and impact frames (Americans, blacks only, or whites only) containing equivalent information. However, the social comparison frame had an unintended effect of *lowering* the risk perception for the group at lower risk (i.e., whites in the case of STDs). Dunham et al. (2016) investigated whether messages about HIV and diabetes using a social comparison frame would increase risk perceptions among blacks (the group at higher risk) relative to non-comparative control messages that did not mention race. The messages using a social comparison frame did not significantly increase risk perceptions among blacks, compared with the non-comparative messages.

Other studies examined the effects of social comparison frames in the context of cardiovascular disease and obesity. Jones et al. (2016) found that public service announcements (PSAs) using a black-white social comparison frame for presenting cardiovascular disease risk negatively affected task persistence (i.e., completing a health assessment form), especially among blacks, relative to PSAs on neutral health topics (air pollution, forest fires, and wearing seatbelts). Skurka (2019) examined the response to obesity messages that used racial (blacks at higher risk than whites) and geographic (rural individuals at higher risk than urban individuals) social comparison frames. Participants exposed to the racial comparison frame were more likely to accept the accuracy of the information than participants in the non-comparative control condition (i.e., participants who received a message that referenced only “adults”). However, the racial comparison frame had negligible effects on other measures of believability (e.g., agreement that the message is credible, counterarguments to the message), emotions, attributions of responsibility, or policy support, relative to the non-comparative frame. Similarly, relative to the non-comparative frame, the geographic comparison frame decreased the perceived credibility of the message and increased message counterarguing, which was associated with less support for obesity prevention policies.

##### Social comparison vs. progress and impact frames

Some studies compared the effects of information presented using a social comparison frame to information presented using a progress frame (Nicholson et al., 2008; Landrine and Corral, 2015; Langford et al., 2017; Lee et al., 2017). A few studies also included comparisons to information presented using an impact frame (Nicholson et al., 2008; Uhrig et al., 2013).

Landrine and Corral (2015) examined reactions to mock news articles about colon cancer and found that within the group at higher risk (i.e., blacks), exposure to social comparison-framed articles did not increase perceived cancer risk or intention to get screened as compared with exposure to an article using a progress frame, which emphasized a decrease in colon cancer death rates in the black community. Uhrig et al. (2013) compared messages about STD disparities that used a social comparison frame (blacks affected by gonorrhea at higher rates than whites), a progress frame (gonorrhea among blacks has declined over past decade), or an impact frame (gonorrhea affects blacks at a high rate). The progress-framed message was most effective in terms of emotional reaction (less upsetting, more encouraging), and the impact-framed message was most effective in motivating participants to want to get tested for STDs and to talk to family and friends about getting tested.

Langford et al. (2017) compared the effects of a social comparison-framed message (“African Americans die from chronic diseases like diabetes at a much higher rate than whites”) with a progress-framed message (“African Americans are increasing their exercise levels”) on willingness to participate in a diabetes prevention and physical activity study and found that message framing had no effect on blacks’ willingness to participate in the study. However, this study did not assess the effects of framing on risk perceptions, emotional reactions, or behavior.

##### Etiological or causal framing

People often have preexisting beliefs about cause and responsibility for a health condition that can vary by race, gender, income, and age (Brady, 2016). These beliefs about cause and responsibility can be influenced by the way a message is framed. Etiological framing can influence perceptions of responsibility and support for policies to reduce health disparities (Niederdeppe et al., 2008). For example, one study compared the effects of four frames addressing varied causes of anorexia nervosa and found that how the message was framed influenced beliefs about the cause of the condition (Bannatyne and Abel, 2015). The condition was framed as being caused by either biology/genetics, sociocultural factors (e.g., media influence, body image ideals), environmental factors (e.g., sporting pressure, modeling of diet behaviors, trauma), or multiple factors (i.e., the interaction between biological, societal, and environmental factors). Participants who received the biological/genetic frame were more likely to attribute the cause to biology and genetics, those who received the sociocultural frame were more likely to attribute the cause to sociocultural factors, and so on. However, the frames also generated some unexpected effects. Participants in the sociocultural and multiple factors conditions believed individuals to be more responsible and blameworthy for their condition than participants in the other conditions. The authors concluded that attributing the cause to biology and genetics may decrease the level of blame and stigma associated with the condition because biology and genetics are factors over which people have no control.

A common etiological frame used in public health messaging is the individual responsibility frame, which emphasizes factors over which individuals have control, such as behaviors that may increase one’s risk of acquiring or developing a disease or health condition (Guttman and Salmon, 2004; Dunham et al., 2016; Lee and An, 2016). A content analysis of video and print PSAs on a variety of health topics found that 80% used an individual responsibility frame (Coleman and Hatley Major, 2014).

A series of experiments examined message framing in mock news articles about being overweight or obese, comparing an individual responsibility frame (i.e., they described being overweight or obese as controllable and inherently unhealthy, and stigmatization and discrimination as acceptable) to a multiple factors frame (i.e., they described being overweight or obese as uncontrollable and not inherently unhealthy, and stigmatization and discrimination as unacceptable; Frederick et al., 2016). These descriptions were based on frames commonly used in news articles about obesity. The researchers found that participants who read articles using an individual responsibility frame expressed more belief in the health risks of being overweight, more belief that weight is controllable, more support for charging obese people more for health insurance, more prejudice against being overweight, more willingness to discriminate against overweight people, and less willingness to celebrate body size diversity. However, they found little or no effect of the individual responsibility frame on support for public policies.

Dunham et al. (2016) found no effect of the individual responsibility frame in messages about HIV and diabetes on risk perceptions, emotional responses, or support for public policy compared with other frames. This study also hypothesized that a combined individual responsibility and social comparison frame would induce denial among the group at higher risk (i.e., blacks) and reduce risk perceptions. Contrary to this hypothesis, a diabetes message using the combined social comparison/individual responsibility frame significantly increased perceived risk among blacks as compared with the control condition. However, an HIV message using this framing did not significantly influence perceived risk among blacks.

### Potential Unintended Adverse Effects of Messages Communicating About Health Disparities

Messages about health disparities can have unintended adverse effects both in the short and long term and at the individual and societal levels. The Institute of Medicine (2002) and others have argued that consideration of unintended adverse effects and other ethical issues in health communication is imperative for both moral (i.e., adhering to ethical principles) and practical (i.e., producing the desired impact) reasons. Messages need to balance the potential benefits of presenting health disparities information to raise awareness and promote behavior change with the potential harms, such as stereotyping and stigmatization (Institute of Medicine, 2002; Guttman and Salmon, 2004; Coleman and Hatley Major, 2014; Keller et al., 2014). For example, while targeting is used to increase the relevance of messages to a priority audience, presenting information that links a particular high-risk group with a negative health condition (i.e., highlighting health disparities) can stigmatize the priority audience (Guttman and Salmon, 2004; Friedman et al.’s, 2014) and cause them to perceive messages as reinforcing stereotypes (Sorhaindo et al., 2016). Next, we discuss literature addressing our second RQ on the potential unintended adverse effects of communicating about health disparities, including stigmatization and stereotyping, victim blaming, negative emotional reactions, mistrust of health information, and boomerang effects.

#### Stigmatization and Stereotyping

In stigmatization, certain attributes become associated with negative evaluations and stereotypes that are well-known in a community or culture and become the basis for excluding or avoiding members of the stereotyped group (Major and O’Brien, 2005). Health messages can inadvertently stigmatize and stereotype people based on their health-related behaviors (e.g., smoking, sexual behavior) or health condition (e.g., HIV). These effects are not benign, as they can affect the identity of individuals and groups and influence the way people perceive themselves and how they are perceived by others (Guttman and Salmon, 2004; Guttman, 2017). Stigmatized individuals may be feared, avoided, regarded as de*via*nt, or blamed for their health condition.

Messages that use a social comparison frame can activate a stereotype threat response in individuals exposed to the message, a phenomenon in which these individuals perceive that they are at risk of confirming negative stereotypes about their group (Cho and Salmon, 2007; Inzlicht and Schmader, 2011; Lee et al., 2017). Stereotype threat is the resulting sense that one might be judged in terms of negative stereotypes about one’s group instead of on personal merit. Researchers hypothesize that stereotype threat can adversely affect the attitudes, intentions, and behaviors of the stereotyped group (Inzlicht and Schmader, 2011).

Evidence of these types of unintended effects include anti-tobacco campaigns that stigmatize smokers and people with smoking-related illnesses (Bayer, 2008; Riley et al., 2017). Patients with lung cancer report feeling stigmatized because of the association with a behavior (smoking) that is perceived to be personally controllable (Chambers et al., 2012; Shen et al., 2016). Lung cancer stigma is associated with negative psychosocial and medical outcomes, including delayed diagnosis, poor quality of life, and poor patient-provider communication (Riley et al., 2017). Additionally, PSAs addressing eating disorders can lead to more negative attitudes and less willingness to interact with individuals with this health condition (Iles et al., 2016, 2017).

In the context of HIV, research suggests that messages about pre-exposure prophylaxis (PrEP) may contribute to stereotypes and stigma associated with PrEP users (Thomann et al., 2018). For example, in focus groups with MSM and transgender women, some participants expressed negative views of those who use PrEP. Participants suggested that their perceptions of who would benefit from PrEP were derived from PrEP marketing campaigns. They described messages about the benefits of PrEP as contributing to the stereotype that those who use PrEP engage in condomless sex with multiple partners (Thomann et al., 2018). Many participants also said that this negative stereotype and associated stigma influenced willingness to use PrEP.

Populations at higher risk for a stigmatized health condition may oppose health communication interventions that present disparities information because of concern about stigmatization and stereotyping (Friedman et al.’s, 2014; Drumhiller et al., 2018). A qualitative study with blacks explored perceptions of STD disparities in the black community and found that participants were reluctant to have STD-related disparities information disseminated to non-black communities. Participants expressed concern that the information would stigmatize blacks, perpetuating racism, and discrimination (Friedman et al.’s, 2014).

##### Priming stereotypes

Another way in which health messages can perpetuate stereotyping is through the process of priming, which can then influence how people are perceived and the judgments made about them (Power et al., 1996; Roskos-Ewoldsen et al., 2009). Priming refers to the automatic activation of representations or associations in memory by exposure to a stimulus, such as a message, which then influences subsequent judgments and behavior (Bargh and Chartrand, 1999; Roskos-Ewoldsen et al., 2009).

Stereotypes can be primed through the personality traits and other characteristics used to describe individuals (Power et al., 1996; Wang, 2019). Depicting individuals in a small number of stereotypical roles or personality types can prime stereotypes and communicate a message quickly, but can also perpetuate those stereotypes (Wang, 2019). On the basis of a study of stigma and counter-stigma frames, cues, and exemplifications in news coverage of depression, Wang (2019) advised that the use of exemplars (i.e., illustrative cases) can be problematic. Stereotypical exemplars can bias judgment and lead tofferroneous generalizations.

Public health messages often use visual images to capture attention, reflect the priority audience, and increase perceptions of the message’s relevance. However, visual images can prime stereotypes about race, gender, or other group identities (Guttman and Salmon, 2004; Coleman and Hatley Major, 2014; Young et al., 2016). Examples include portraying blacks as athletes and women as mothers. Stereotypes can also be primed *via* cues in the image (e.g., the setting); by emphasizing norms frequently associated with a group or culture, and by music; such as the use of hip-hop in an advertisement targeting a black audience. A content analysis of PSAs on various health topics found that racial and cultural stereotyping primes were present twice as often in visual images than in words (7 and 3%, respectively) (Coleman and Hatley Major, 2014). The content analysis also found blacks were disproportionately represented in HIV-related PSAs; more than half (52.5%) of these PSAs featured blacks.

In a study that tested message concepts for a Centers for Disease Control and Prevention (CDC) HIV testing campaign, black women found a message concept, which was designed to promote HIV testing among black women, to be offensive. They noted that the image called for “women from all walks of life” to get tested, yet the image depicted only black women (Uhrig et al., 2017). Similarly, Drumhiller et al. (2018) examined receptivity to HIV testing campaign messages and found that the participants (black and Hispanic/Latino MSM) objected to images of gay men perceived to be stereotypical (e.g., flamboyant, excessively feminine). The participants reported that stereotypical images of gay men and cues such as the location of campaign materials in “at-risk” neighborhoods made them feel stigmatized because of their race and sexual identity. Images can also influence estimates of rates of disease for specific racial or ethnic groups. In one study, the inclusion of a photograph of a person from a specific racial or ethnic group led to higher estimates of disease risk for that group, even though the text provided no information about the relative risk of disease by race or ethnicity (Gibson and Zillmann, 2000).

Research has consistently found that people tend to remember visual images better than words, referred to as the “picture superiority effect” (McBride and Anne Dosher, 2002). Consequently, the use of images to support frames and the potential for images to prime stereotypes or to promote stigmatization should be carefully scrutinized (Coleman and Hatley Major, 2014). One study that provides support for this conclusion showed participants messages with stigmatizing images of overweight people or non-stigmatizing images and text that emphasized individual or social determinants of obesity (Young et al., 2016). The results revealed a stronger effect of images compared to text. Stigmatizing images influenced behavioral intention among normal-weight participants, even when the text pointed to social determinants. The researchers suggested that the stigmatizing images may have primed an avoidance response in normal-weight participants such that they shifted their behavioral intentions to avoid the stigmatized condition. However, message condition had no effect on the behavioral intentions of overweight participants. The study did not measure emotional response, so it is unknown whether the stigmatizing messages elicited negative emotion or reinforced self-stigma in overweight or obese individuals.

Stereotypic portrayals can influence perceptions about responsibility (Power et al., 1996). One study tested the effects of stereotypic and counter-stereotypic portrayals of blacks and women on attributions or responsibility and perceptions of credibility (Power et al., 1996). The results showed that negative stereotypic portrayals of blacks resulted in more internal or personal attributions of responsibility in subsequent judgments made about blacks. In contrast, positive counter-stereotypic portrayals generated more external or situational attributions of responsibility in subsequent judgments. Stereotypic portrayals of women decreased the perceived credibility of women, whereas counter-stereotypic portrayals increased perceptions of women’s credibility. Similarly, another study found that an article depicting suicidal individuals as outgroup members by describing them in stereotypic terms (e.g., insane, unemployed, juvenile delinquents) generated more stigma than an article describing suicidal individuals as ingroup members (e.g., anyone; Lee and An, 2016).

#### Victim Blaming

Health messages framed in terms of individual responsibility can result in victim blaming—identifying the cause of the health problem as being the result of an individual’s behavior without recognition of social and environmental forces (Institute of Medicine, 2002; Guttman and Salmon, 2004; Cho and Salmon, 2007; Coleman and Hatley Major, 2014; Riley et al., 2017). Linking health with personal responsibility may, by implication, characterize individuals who do not adopt recommended health behaviors as weak or irresponsible. People may react to these types of messages with feelings of guilt, shame, or frustration when they feel they cannot adopt the recommended health behaviors.

In addition to the potential negative emotional effects of presenting information about health disparities using an individual responsibility frame, this frame frequently does not impart a complete understanding of the causes of a disease or condition. In some cases, individual behavior may not actually be responsible for the existence of a disparity. For instance, a disparity in the rate of HIV infection among black MSM compared with other MSM is not the result of black MSM engaging in risky sexual behaviors at higher rates than MSM generally (Matthews et al., 2016). Instead, the disparity in HIV infection rates is the result of a variety of complex, interrelated factors (McCree et al., 2017).

#### Negative Emotional Reactions

Messages sometimes use negative emotion (e.g., fear, guilt) to communicate a health risk associated with a particular group or identity (Coleman and Hatley Major, 2014; Fairchild et al., 2015). Examples include New York City’s fear-based tobacco, obesity, and HIV health communication campaigns (Fairchild et al., 2015). An analysis of PSAs on health topics found that the use of negative emotion was the second-most common frame used in PSAs—present in 48% of the sample—after individual responsibility (Coleman and Hatley Major, 2014).

Social comparison framing can elicit counterproductive negative emotional reactions among the population at higher risk. Several studies compared emotional reactions to messages presented using a social comparison frame as compared with other frames. Uhrig et al. (2013) found that communicating about disparities in STD rates among blacks using a social comparison frame was more upsetting and less encouraging relative to using either a progress or impact frame. In another study, blacks exposed to mock news articles about disparities in colon cancer using a social comparison frame (blacks are doing worse than whites) experienced more negative emotional reactions than those exposed to articles using a progress frame (blacks are improving over time) or impact frame (colon cancer strikes blacks at a high rate) (Nicholson et al., 2008). Landrine and Corral (2015) also examined emotional reactions to news articles about disparities in colon cancer and found that blacks exposed to a social comparison frame felt more insulted, discouraged, and angry compared with those exposed to a progress frame.

Lee et al. (2017) examined the effects of messaging about health disparities in the lesbian, gay, bisexual, and transgender (LGBT) community. Participants exposed to the message presented using a social comparison frame reported that it made them feel discouraged, insulted, angry, and significantly less likely to indicate pride in their LGBT identity, relative to the progress-framed message (Lee et al., 2017). When study participants (black men and women) were informed about racial disparities in STD rates in their community within the context of a qualitative study, they often reacted with surprise, sadness, fear, and despair (Friedman et al.’s, 2014).

#### Mistrust of Health Information

Social comparison framing may increase distrust of health information among the population at higher risk. This is an important concern given the prevalence of medical mistrust among racial and ethnic minorities, which has been found to influence attitudes and behaviors related to HIV prevention and treatment (Bogart et al., 2010; Mimiaga et al., 2016; Cahill et al., 2017; Thomann et al., 2018). For example, among black men, mistrust of PrEP is a barrier to use (Cahill et al., 2017; Thomann et al., 2018), and belief in conspiracy theories about antiretroviral therapy is related to treatment non-adherence (Bogart et al., 2010).

Several studies found that social comparison framing was associated with higher distrust, compared with other types of framing. In one study, blacks exposed to news articles about disparities in cancer risk, using a social comparison frame, had more doubts about the veracity of the articles (i.e., they were more likely to agree with the statement, “I wonder if it’s true. I am suspicious of the story”), compared with those exposed to non-comparative articles (Landrine and Corral, 2015). Lee et al. (2017) found that study participants exposed to a message using a social comparison frame (“With rates double that of the population, smoking poses a deadly threat to the LGBT community”) or impact frame (“Half of black gay men will get HIV in their lifetime”) had lower agreement with the statement “I believe the message” than those exposed to a message using a progress frame (“LGBT communities are working to address health problems”).

Dunham’s study (Dunham et al., 2016) of HIV and diabetes messages found that blacks were significantly *less* likely to trust the accuracy of “government data” about racial disparities in HIV prevalence when the information was presented with an individual responsibility frame relative to a non-comparative frame (control group). Conversely, white participants were significantly *more* likely to trust “government data” when presented with a social comparison frame. The findings were mixed for effects of the individual responsibility frame on white participants; this frame significantly decreased trust in the HIV message but significantly increased trust in the diabetes message.

Friedman et al.’s qualitative study (Landrine and Corral, 2015) of perceptions of STD disparities among blacks found that although most participants believed the information, some were skeptical. These participants questioned the objectivity of data sources, suggested the government may inflate or fabricate rates to encourage people to get tested, or disbelieved the lower rates of STDs reported for other racial groups.

#### Boomerang Effects

A well-recognized unintended consequence of health communication messages is the boomerang effect, which refers to health messages having an effect opposite of the intended effect (Cho and Salmon, 2007). For example, obesity-related messages perceived as stigmatizing have been found to result in increased calorie consumption and decreased motivation to lose weight (Schvey et al., 2011; Puhl et al., 2013; Major et al., 2014; Young et al., 2016).

Health disparities information may have a boomerang effect if the group at higher risk avoids, devalues, or rejects the information. People may not believe, or may view as prejudiced, information threatening their self-concept or favorable image of their group. Social comparison-framed messages about cancer disparities can have this type of unintended effect (Nicholson et al., 2008). Blacks exposed to mock news articles about colorectal cancer mortality experienced more negative reactions to articles using a social comparison frame compared with those exposed to articles using a progress frame or impact frame, and they were less likely to have screening intentions. Medical mistrust moderated this effect, with the progress-framed articles producing higher intentions to get screened than the social comparison-framed articles among participants with a high level of mistrust. Participants with a low level of mistrust did not differ in terms of their screening intentions across conditions.

## DISCUSSION

**FIGURE 1 |:**
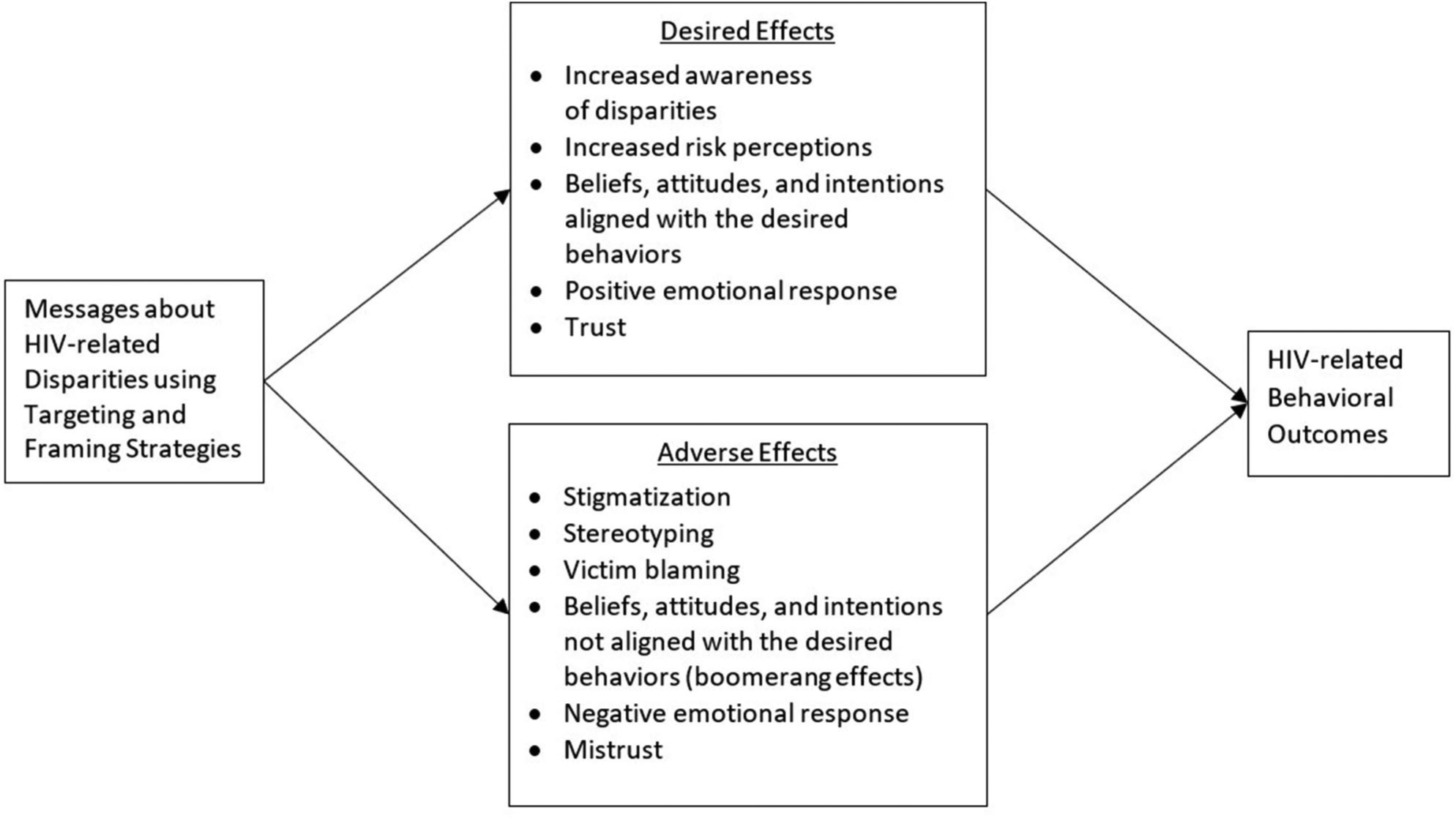
Positive and negative effects of communication about HIV-related disparities.

Based on our review of the broader body of literature addressing the effects of messages about health disparities, we developed a conceptual framework that presents potential positive and negative effects of communication about HIV-related disparities (see Figure 1). Although we hypothesize responses to messages about health disparities to be similar across health contexts, this model will need to be tested empirically in the context of HIV. Next, we discuss implications of the literature that we reviewed and offer recommendations for communicating about HIV-related disparities based on the available evidence. We conclude by proposing a research agenda to fill gaps in the evidence base regarding effective strategies for communicating about disparities across the HIV continuum.

The goal of this literature review was to identify promising approaches for effectively communicating about health disparities across the HIV care continuum. Given our RQs, we specifically intended to examine strategies used to communicate health disparities and investigate potential unintended adverse effects of messages communicating health disparities to identify how specific message strategies contribute to the unintended effects. We found limited literature specifically addressing the RQs in the context of HIV. Although there is a substantial body of literature on communication interventions that address HIV (e.g., Noar et al., 2009), the body of research focused on examining the effects of messages communicating HIV-related disparities within and outside priority audiences is limited. There are likely multiple explanations for why this body of research is not more developed. One possible explanation may be that focusing on social determinants of health and the social and environmental processes and inequities that contribute to health disparities is still relatively recent in the U.S. (Braveman and Gottlieb, 2014). Increasing attention to these factors has recently contributed to the interest in providing higher-risk groups with more context to help them better understand underlying reasons for the disparities. Additionally, disparities in HIV-related outcomes between some groups, such as blacks and whites, have continued to increase (Allgood et al., 2016). Recent research has also begun to highlight a growing concern that messages about HIV-disparities may have unintended effects (e.g., Lee et al., 2017; Thomann et al., 2018). Thus, the aim to increase awareness of these disparities while avoiding unintended effects has become more crucial over time.

A common strategy for communicating about health disparities is to use a social comparison frame, which compares the differences in rates of disease or outcomes between a group more at risk and a group less at risk. Although this is an intuitive strategy for attempting to increase risk perceptions within a priority audience, which is an important predictor of health behavior, studies often find that a social comparison frame does not increase risk perceptions in the group more at risk (Bigman, 2014; Landrine and Corral, 2015; Dunham et al., 2016). Additionally, the evidence points to several potential adverse consequences that can occur with social comparison framing, including stigmatization and stereotyping, negative emotional reactions, and distrust of the information (Landrine and Corral, 2015; Lee et al., 2017).

When considering including direct comparisons between racial or other subgroups, as is often done in messages that communicate health disparities, it is important to understand how social psychological processes might influence message effects. Social comparison-framed messages can be perceived as a threat to one’s group and individual identity. Social identity theory provides a framework for understanding the relationship between social comparison and intergroup processes (Tajfel, 1982). For a group that suffers from a lower status in society, direct comparisons with a higher-status group can have negative psychological consequences, such as devaluing one’s group, engaging in self-hate, and expressing preferences for the outgroup. Alternatively, because people are motivated to maintain a positive social identity and self-image, for members of the lower-status group, social comparisons can also result in attributing the cause of the discrepancy to external factors that are outside of one’s control and can generate greater ingroup/outgroup distinctions and ingroup favoritism. Social comparisons also can increase outgroup bias (i.e., negative evaluations of outgroup members) among members of the higher-status group. It is easy to see how these responses are counterproductive to the goals of messages designed to communicate about health disparities and can have detrimental individual and societal effects.

Another framing strategy, the individual responsibility frame, addresses health disparities by emphasizing the role of individuals in both increasing and reducing their risk. This approach can generate negative emotional responses, reinforce stigma, and result in distrust of the information (Dunham et al., 2016). It also places the responsibility for health disparities on the individual, when this is often not accurate (Matthews et al., 2016). Another challenge with this frame is that people have preexisting beliefs about groups and health risks that can influence how they process and respond to messages (Brady, 2016; Calabrese et al., 2016; Thomann et al., 2018). For example, research in social psychology has identified biases in how social information is processed. One such bias, known as the fundamental attribution error, reflects a tendency when making causal judgments to overestimate the influence of personal factors and underestimate the influence of environmental factors (Ross, 1977). Consequently, messages that focus on individual responsibility as a causal explanation for health disparities serve to reinforce rather than challenge psychological biases.

Communicating about health disparities also has the potential to prime stereotypes *via* the use of a variety of visual and textual cues. Visual images are particularly influential, and they have the potential to overpower text, reinforce stereotypes, and perpetuate stigma (McBride and Anne Dosher, 2002; Coleman and Hatley Major, 2014; Young et al., 2016; Uhrig et al., 2017).

Thus, messages about health disparities share many of the characteristics that Smith (2007) described as being present in messages that communicate stigma. They draw attention to distinct groups of people defined by racial, social, or behavioral characteristics; they link these groups to a physical threat (i.e., HIV or another health condition); and by using either a comparative or individual responsibility frame, or in some cases both, they suggest indirectly or directly that group members are responsible for the threat. Although the intention behind messages about health disparities is to increase awareness and motivate positive behavior change, the characteristics of these messages can instead generate unintended effects for both the unstigmatized group—including social distancing, negative attitudes, and support for stricter policies—and the stigmatized group.

Unintended effects—such as negative emotional reactions, decreased trust in health information, perceptions of blame, and stereotyping—can cause members of the priority audience to distance themselves from and reject messages about health disparities. Rather than reducing risk and improving health outcomes, these messages can worsen health if they backfire and can also have negative psychological consequences for members of the priority audience. These potential iatrogenic effects are especially important to consider when communicating about HIV-related disparities, as the same groups that experience disparities across the HIV care continuum also experience intersectional stigma (Earnshaw et al., 2013; Rice et al., 2018), which could be further exacerbated by the way disparities information is communicated. In addition, medical mistrust among racial and ethnic minorities has been found to influence attitudes and behaviors related to HIV prevention and treatment (Bogart et al., 2010, 2011; Cahill et al., 2017; Thomann et al., 2018), and this mistrust could also be perpetuated by the framing of disparities information. As such, messages need to balance the potential benefits of communicating HIV disparities to raise awareness and promote behavior change with the potential harms that may result from the framing (Institute of Medicine, 2002).

It is also critical to ensure that members of priority audiences are involved in message development, pretesting, and implementation of communications. Involving members of the priority audience in these activities is best practice in public health communication. However, it also serves to empower communities that face systemic inequities and foster collective action to reduce disparities and improve health outcomes (Douglas et al., 2016; Thompson et al., 2016). Messages and communications that address health disparities with the intention of fostering individual and community empowerment can shift the focus from individual blame to a fuller understanding of the multi-level factors that contribute to health disparities (Douglas et al., 2016). This approach also has the potential to lead to more effective messages and interventions by increasing trust and credibility among the priority audience (Earnshaw et al., 2013).

### Recommendations and Agenda for Future Research

Applying the available evidence on message framing to HIV, we offer recommendations for communicating about HIV-related disparities, presented in Table 3.

Some evidence suggests that using a progress frame to present health disparities information may be more likely to generate positive emotional and behavioral responses than using a social comparison frame (Nicholson et al., 2008; Landrine and Corral, 2015; Lee et al., 2017). However, the evidence comprises only a handful of studies—some of which compared only a progress frame with a social comparison frame—and even though the progress frame performed better, the extent of its positive effect is unclear. In addition, none of the studies were specific to HIV. Other approaches to message framing, such as using an impact frame, may also be effective for communicating about HIV-related disparities (Uhrig et al., 2013). Furthermore, it is unclear which message framing strategy is most effective when communicating about HIV-related disparities to the general public vs. targeting messages to subpopulations at high risk for getting or transmitting HIV.

Due to the limitations in the existing body of evidence, we propose a research agenda to examine strategies for effectively communicating about HIV-related disparities, while avoiding unintended effects (Table 4).

## CONCLUSION

Health communication can play an important role in reducing HIV-related disparities and stigma, which is a central priority of *Ending the HIV Epidemic: A Plan for America* (U.S. Department of Health and Human Services, 2020). Further efforts are needed to develop and test communication strategies capable of raising awareness of, influencing attitudes and beliefs about, and motivating behavior change necessary to reduce HIV-related disparities without resulting in stigmatization or other unintended adverse effects.

## Figures and Tables

**TABLE 1 |:** Literature search overview.

	Database search	Snowball method
Time period	2011–2018	1996–2018
Language	English only	English only
Location	U.S. focus	U.S. focus
Sources	PubMed Web of Science (includes Science Citation Index Expanded, Social Sciences Citation Index, and Conference Proceedings Citation Indexes for Science and Social Science & Humanities) PsyclNFO Communication Source	Reference lists of relevant published articles Colleagues with subject matter expertise
Keywords (first level)	[“message framing” or “priming” or “targeting” or “health message” or “health communication” or “health information” or “public service announcement” or “campaign”] AND [“health disparities*” or “racial disparities*” or “health equity*” or “racial stigma*” or “stigma* or “stereotype” or “intergroup differences”] OR [“health risk estimates*” or “health risk*” or “risk*”] OR [“perceived susceptibility*” or “perceived risk*”]; **AND**	Not applicable
Keywords (second level)	[“Black” or “African American”] or [“Hispanic” or “Latino”] OR [“MSM” or “men who have sex with men”] OR [“homosexual” or “gay” or “bisexual”] OR [“transgender” or “transsexual”] OR [“minority”] or [sexual minority or gender minority]; **OR**	Not applicable
Keywords (third level)	[“unintended effects” or “unintended consequences” or “iatrogenic effects”] OR [“ethical considerations”]	Not applicable
Publications and other documents worthy of full-text review	39	50
Excluded because of a lack of relevant information	19	27
Total publications and other documents reviewed	20	23

**TABLE 2 |:** Overview of studies testing message framing effects.

Study	Health topic(s)	Study population	Message frames compared	Framing effects
Bannatyne and Abel (2015)	Anorexia nervosa	University students (76% female)	Four etiological (i.e., causal) frames:	**Perceptions about responsibility**
			Biology/genetics Sociocultural factors (media influence, body image ideals) Environmental factors (sporting pressure, modeling of diet behaviors, trauma) Multiple factors (interaction between biological, societal, and environmental factors)	Multifactorial condition increased perceptions that individuals were responsible for their condition compared with the biological/genetic and environmental conditions Multifactorial and sociocultural conditions increased perceptions that individuals were to blame for their condition compared with the other conditions
Bigman (2014)	Sexually transmitted	Black, white	Study 1:	**Risk perceptions**
	infections (STIs)		Social comparison Impact (black or white risk only) Non-comparative	Social comparison frame did not increase perceived risk for the more at-risk group (blacks) compared with impact (black risk only) or non-comparative frames Social comparison frame produced lower perceived risk for less at-risk group (whites) compared with impact (white risk only) frame
	Skin cancer	Black, white	Study 2:	**Risk perceptions**
	(incidence)		Social comparison Impact (black or white risk only) Non-comparative	Social comparison frame did not increase perceived risk for more at-risk group (whites) compared with impact (white risk only) and non-comparative frames Social comparison frame produced lower perceived risk for less at-risk group (blacks) compared with impact (black risk only) and non-comparative frames
	Skin cancer (survival)	Black, white	Study 3:	**Risk perceptions**
			Social comparison Impact (black or white risk only) Non-comparative	Impact (white risk only) and non-comparative frames produced higher perceived risk for less at-risk group (whites) compared with the social comparison frame Social comparison frame produced higher perceived risk for the higher-risk group (blacks) compared with impact (white risk only) and non-comparative frames
Dunham et al. (2016)	HIV/AIDS Diabetes	Black, white	Social comparison Individual responsibility Non-comparative/does not emphasize individual responsibility (control)	**Risk perceptions**
				**Among blacks:**
				No difference in risk perceptions between social comparison and non-comparative frames (HIV and diabetes) Risk perceptions were higher in combined social comparison and individual responsibility frame than control (diabetes)
				**Among whites:**
				Risk perceptions were lower in individual responsibility frame than control (HIV) Risk perceptions were higher in individual responsibility frame and combined social comparison/individual responsibility frame conditions than control (diabetes)
				**Perceived credibility**
				**Among blacks:**
				Lower level of trust in information for individual responsibility frame compared with control (HIV)
				**Among whites:**
				Higher level of trust in information in social comparison frame than control (HIV and diabetes) Lower level of trust in information in individual responsibility frame than control (HIV)
Frederick et al. (2016)	Overweight and obesity	Consisted of university students and participants recruited from Mechanical Turk	Individual responsibility Outside of one’s control	**Risk perceptions** Individual responsibility frame increased perceptions of the risks of being overweight/obese compared with other frame **Other effects** Individual responsibility frame produced greater belief that weight is controllable, more support for charging obese people more for health insurance, more prejudice against overweight people, more willingness to discriminate against overweight people, and less willingness to celebrate body size diversity compared with other frame
Jones et al. (2016)	Cardiovascular disease risk	Black	Social comparison + neutral health topics Neutral health topics	**Behavior** Social comparison frame reduced task persistence (completing a health self-assessment)
Landrine and Corral (2015)	Colon cancer	Black	Social comparison Non-comparative	**Risk perceptions** No difference in perceived cancer risk **Behavioral intentions/behavior** No difference in intention to get screened for colon cancer or to recommend screening for family **Emotional reactions** Social comparison frame produced more negative response (insulted, discouraged, angry, suspicious) compared with non-comparative frame
Langford et al. (2017)	Diabetes	Black	Social comparison Progress	**Behavioral intentions** No difference in intention to participate in diabetes prevention study
Lee et al.(2017)	General health problems, HIV, and smoking	LGBT	Social comparison Progress Impact	**Emotional reactions** More negative responses (discouraged, insulted, and angry) to social comparison frame than progress and impact frames More positive responses (hopeful, feel good, proud, inspired and encouraged) to progress frame than social comparison or impact frames **Perceived credibility** Higher perceptions of message credibility in progress condition than social comparison or impact conditions
Nicholson et al. (2008)	Colorectal Cancer	Black	Social comparison Impact Progress	**Behavioral intentions** Progress frame produced increased desire to be screened compared with impact or social comparison frames **Emotional reactions** Progress frame produced more positive response compared with impact or social comparison frames Social comparison frame produced more negative response compared with impact or progress frames
Skurka (2019)	Obesity	Recruited through Mechanical Turk (82% white, 10% black)	Social comparison (racial comparison) Social comparison (geographic comparison) Non-comparative	**Emotional reactions** No difference in responses (sympathy, anger) between the racial comparison frame or geographic comparison frame and the control **Perceived credibility** Higher acceptance of the accuracy of the information in the racial comparison compared with the control condition. No difference between the racial comparison frame and control on other measures of believability (agreement that the message is credible, counterarguing the message), attributions of responsibility, or policy support Lower perceived credibility of the message and increased message counterarguing in geographic comparison compared with control condition
Uhrig et al. (2013)	STD (gonorrhea)	Black	Social comparison Progress Impact	**Risk perceptions** Social comparison and impact frames generated greater agreement with the statement “Gonorrhea rates are high among African Americans” than the progress frame **Behavioral intentions/behavior** Impact frame more likely than other frames to motivate participants to want to get tested for STDs and to talk to family and friends about getting tested **Emotional reactions** Progress frame less upsetting and more encouraging than other frames **Perceived credibility** Trust in information higher in impact than social comparison condition

**TABLE 3 |:** Recommendations for communicating about HIV disparities.

✓ Use progress framing and appeals that evoke positive emotions that motivate action (e.g., hope, encouragement, positive roles) rather than messages that evoke sadness and can be demotivating (Lazarus, 1991; Nicholson et al., 2008; Friedman et al.’s, 2014; Landrine and Corral, 2015; Frederick et al., 2016).
✓ Address distrust in disparities information by ensuring data are transparent and presented credibly (Nicholson et al., 2008). For example, include verifiable sources of information, such as a publicly accessible website, and information about data collection and how rates are derived.
✓ Recognize social and societal factors that contribute to HIV disparities while also motivating individuals to “take charge” (e.g., adopt specific behaviors) by including a strong efficacy message regarding what actions individuals have the power to take (Lundell et al., 2013; Friedman et al.’s, 2014). It may also be useful to take a social justice approach within messages, focusing on resiliency—at both the individual and community levels—as a means to address disparities (Matthews et al., 2016).
✓ Use images and exemplars strategically to avoid reinforcing stereotypes (Coleman and Hatley Major, 2014). Pretest images with members ofthe target audience to ensure they are not offensive (Uhrig et al., 2017).
✓ Carefully consider the use of cultural symbols and themes (Institute of Medicine, 2002). When developing messages, ask the following: Will the use of cultural themes stereotype the population? Are there individuals or groups that may be excluded or stigmatized when cultural themes are a dominant part of the communication intervention? Are cultural symbols or themes used in a messsage relevant to the advocated behavior (as identified through formative research), or do they represent outside perceptions of what may be valued or familiar to the audience (i.e., stereotypes)?

**TABLE 4 |:** Agenda for future research on communicating about HIV disparities.

Research activity	Rationale
Experimentally compare messages about HIV-related disparities across the continuum of care using progress, impact, and social comparison frames.	Social comparison-framed messages about health disparities do not always increase risk perceptions in the target audience (Bigman, 2014; Landrine and Corral, 2015; Dunham et al., 2016) and can have adverse effects, including stigmatization and stereotyping, negative emotional reactions, and distrust of the information (Landrine and Corral, 2015; Lee et al., 2017). Additionally, only one previous study compared framing strategies in the context of HIV (Dunham et al., 2016). Thus, it would be beneficial to examine whether frames other than a social comparison frame can increase risk perceptions without having adverse effects.
Experimentally test message strategies that acknowledge the multiple factors (i.e., social determinants) that contribute to HIV risk and may be outside of an individual’s control, while also acknowledging the role of the individual in reducing risk.	Challenges that emerge from the literature on health disparities messages are that (1) messages that discuss the contribution of social determinants of health may not address individual behavior and thus may not motivate health behavior change (Lundell et al., 2013), and (2) messages that address individual health behavior alone may be perceived as stigmatizing and can perpetuate misunderstanding about the cause of a disparity (Matthews et al., 2016). Thus, it would be useful to investigate whether messages that discuss both social/societal and individual factors reduce negative responses to messages while also motivating behavior change. This is a type of mixed or competitive frame that tends to be overlooked in research on message framing effects (Guenther et al., 2020).
Assess how images and exemplars can be incorporated in HIV disparities messages to increase personal relevance, attention, and persuasiveness without reinforcing disparities related stereotypes. Studies can evaluate different combinations of images, exemplars, and text to assess emotional response and effects on risk perceptions and other outcomes.	Exemplars and images can prime stereotypes and bias judgment (Guttman and Salmon, 2004; Coleman and Hatley Major, 2014; Arpan et al., 2017). Visual images within health messages tend to be more influential than text; although they are often used to increase message relevance, they can be also perceived as stereotypical and offensive by members of the target audience (Uhrig et al., 2017; Drumhiller et al., 2018).
Examine how anti-stigma communication approaches found to be effective in reducing mental health stigma may be used in HIV communication interventions.	Meta-analyses of mental health anti-stigma communication research found that approaches that facilitate interpersonal or “mediated” contact successfully reduced stigma associated with mental illness (Corrigan et al., 2012, 2015). Creative approaches are needed to develop opportunities for “contact” with people with HIV and to evaluate effects on audiences. One way of mediating contact with stigmatized groups is by using photovoice, an approach used to counter stereotypes, external stigma, and internal stigma (Wang et al., 2000; Russinova et al., 2014; Centers for Disease Control and Prevention (CDC), 2019). Although these studies suggest some promise, this approach has not been tested in combination with framing or in the context of HIV
Examine whether messages about HIV disparities designed to elicit positive emotions such as encouragement and hope—similar to progress-framed messages—are effective in motivating positive behavior change.	According to functional theories of emotion in psychology, emotions are elicited in response to our environment and motivate action in ways that are consistent with personal goals (Lazarus, 1991). This perspective on emotion suggests that the response to shame, which is associated with stigma and the perception that one is being stereotyped, is to hide and avoid facing what may be perceived by oneself or others as personal failure (Lazarus, 1991). As this is not the desired response to health risk messages, other approaches need to be investigated. Two studies that tested responses to skin cancer prevention messages found that hope was positively associated with self-efficacy perceptions and that hope and self-efficacy predicted intentions to engage in skin cancer prevention behaviors (Nabi and Myrick, 2019).
Investigate whether integrating self-affirmation with health risk messages about HIV disparities will be effective in promoting positive behavior change.	Messages that present information about health disparities can be perceived as threatening to one’s social identity (Tajfel, 1979). Previous research found that engaging in a self-affirmation exercise before exposure to a threatening health message can reaffirm one’s self-concept, thus increasing message acceptance and positive health behavior change (Epton and Harris, 2008). A recent study found that a health risk message that incorporated self-affirming text in the message produced greater intentions to reduce risky behaviors (Arpan et al., 2017).
Examine whether messages focused on fostering individual and community empowerment will increase trust and be more effective in generating positive individual- and community-level responses to reduce HIV disparities.	Messages about health disparities can be perceived as blaming individuals for poor health outcomes, which can produce a multitude of adverse effects and make messages ineffective or, worse, harmful. Incorporating community members and their feedback into message development and campaign implementation can increase the likelihood that these interventions will be effective (Earnshaw et al., 2013). It can also increase the capacity of messages and communication campaigns to empower individuals and communities to engage in positive behaviors and actions that reduce health disparities (Douglas et al., 2016; Thompson et al., 2016).
